# An Analysis of Costs and Health Co-Benefits for a U.S. Power Plant Carbon Standard

**DOI:** 10.1371/journal.pone.0156308

**Published:** 2016-06-07

**Authors:** Jonathan J. Buonocore, Kathleen F. Lambert, Dallas Burtraw, Samantha Sekar, Charles T. Driscoll

**Affiliations:** 1 Center for Health and the Global Environment, Harvard T.H. Chan School of Public Health, Boston, Massachusetts, 02215, United States of America; 2 Harvard Forest, Harvard University, Petersham, Massachusetts, 01366, United States of America; 3 Resources for the Future, Washington, District of Columbia, 20036, United States of America; 4 Department of Civil and Environmental Engineering, Syracuse University, Syracuse, New York, 13244, United States of America; CSIRO, AUSTRALIA

## Abstract

Reducing carbon dioxide (CO_2_) emissions from power plants can have important “co-benefits” for public health by reducing emissions of air pollutants. Here, we examine the costs and health co-benefits, in monetary terms, for a policy that resembles the U.S. Environmental Protection Agency’s Clean Power Plan. We then examine the spatial distribution of the co-benefits and costs, and the implications of a range of cost assumptions in the implementation year of 2020. Nationwide, the total health co-benefits were $29 billion 2010 USD (95% CI: $2.3 to $68 billion), and net co-benefits under our central cost case were $12 billion (95% CI: -$15 billion to $51 billion). Net co-benefits for this case in the implementation year were positive in 10 of the 14 regions studied. The results for our central case suggest that all but one region should experience positive net benefits within 5 years after implementation.

## Introduction

In June 2014, the U.S. Environmental Protection Agency (EPA) proposed draft standards for carbon dioxide (CO_2_) emissions from existing power plants–the Clean Power Plan–which were finalized in August 2015[1]. Fossil fuel-fired power plants make up 31% of U.S. greenhouse gas (GHG) emissions, largely CO_2_, and by 2030, the final version of the Clean Power Plan would reduce CO_2_ emissions by 32% below 2005 levels[1]. Reducing CO_2_ emissions from power plants can have public health “co-benefits” by simultaneously decreasing sulfur dioxide (SO_2_), nitrogen oxides (NO_x_)_,_ and primary fine particulate matter (PM_2.5_) emissions, resulting in lower ambient air concentrations of PM_2.5_ and ozone [1–5], and can be an important part of policy decision-making. Driscoll et al. (2015) examined three different scenarios that were available in 2014 for a U.S. Federal standard for CO_2_ emissions from power plants, and simulated the air quality and health co-benefits of these different policy scenarios[2]. Of the three analyzed in Driscoll *et al*. [2], the policy that most resembled the final U.S. Clean Power Plan had the greatest health co-benefits.

Despite the fact that health co-benefits generally represent the largest share of near-term economic benefits associated with climate change mitigation[6,7], few studies have examined both the magnitude and the spatial distribution of costs and co-benefits of such actions. Economic analysis of the Clean Power Plan has thus far considered only partial equilibrium effects [8,9], thereby excluding hidden costs from implicit taxes on factors of production and hidden benefits associated with improved labor productivity from air quality improvements. The U.S. EPA Regulatory Impact Analysis for the Clean Power Plan estimated the partial equilibrium total national costs and benefits, but not in a spatially explicit manner[10,11]. Here, we build on the analysis of air quality and health co-benefits in Driscoll et al.[2] by estimating and mapping co-benefits and costs for 14 power supply regions under the policy scenario that most closely resembles the U.S. Clean Power Plan. We use three different energy efficiency cost cases and a simulated implementation year of 2020. In doing so, we answer the following questions: (1) how do the magnitude of costs and co-benefits change under varying assumptions; (2) how are the costs and co-benefits spatially distributed; and (3) what can we infer about relationship between costs and co-benefits of the policy over time?

## Materials and Methods

### Estimation of Health Co-Benefits

The methods used to estimate the health co-benefits in terms of the number of cases are described in detail in Driscoll et al. (2015)[2] and summarized here. The Integrated Planning Model (IPM)[12], a dynamic power sector production cost linear optimization model of the North American power grid, was used to simulate the power sector response to the carbon standard, and to estimate emissions of CO_2_, SO_2_, NO_x_, and directly emitted PM_2.5_ from 2,417 fossil fuel-fired power plants in the U.S. under a “business-as-usual” (BAU) reference scenario based on the U.S. Energy Information Administration 2013 Annual Energy Outlook [13] and a moderately stringent but highly flexible policy scenario, available in 2014, that resembles the final Clean Power Plan, using 2020 as an implementation year. This policy scenario allows for the use of different compliance mechanisms, including demand-side energy efficiency, efficiency and heat rate upgrades to power plants, power plants co-firing with lower-carbon fuels, electrical grid dispatch to lower-carbon generation, and trading of emissions within and between states. The resulting emissions estimates from the IPM model for this scenario were inputted into the Community Multiscale Air Quality (CMAQ) model v4.7.1[14], using the 12 km x 12 km grid for the continental U.S., to simulate the concentration of PM_2.5_ and ozone under this scenario, and under BAU. The results of the CMAQ runs were input to BenMAP CE v.1.1[15], a Geographic Information System (GIS) model designed to calculate health impacts of air pollution, air quality management scenarios, and other applications. We used BenMAP to estimate the number of cases and distribution of co-benefits for six health outcomes based on the difference between the policy scenario and BAU (Table 1). The health co-benefits in this analysis are conservative and do not include possible benefits from reducing other health effects, such as asthma[16], stroke[17], and autism[18]; benefits associated with decreased emissions of hazardous air pollutants (e.g., mercury)[19]; pediatric benefits[16]; or the direct health benefits of climate change mitigation[20,21]. We use the valuation module in BenMAP CE v1.1[15] with default methods and values to estimate the economic value of the co-benefits at county, power region, and national scales[22–24]. Details on the health impact functions and valuation methods are available in S1 and S2 Tables.

**Table 1:** Health co-benefits of moderately stringent, highly flexible carbon standards by health endpoint for the central estimate and 95% confidence intervals. Estimates are rounded to two significant figures. Monetized values are in 2010 USD.

Health endpoint	Source of Concentration-Response Function:	Health co-benefits (# of cases) (95% CIs)	Health co-benefits (million 2010 USD) (95% CIs)
Mortality, All Cause	Roman et al.[25]	3,200 (680–5,600)	$26,000 ($1,900–$63,000)
Mortality, All Cause	Jerrett et al.[26]	300 (100–500)	$2,500 ($300–$5,700)
Hospital Admission, All Respiratory	Ji et al.[27]	410 (150–680)	$13 ($4.7–$22)
Hospital Admission, All Cardiovascular (except heart attacks)	Levy[28] Zanobetti[29] Pooled	330 (230–440)	$13 ($8.7–$17)
Hospital Admission, All Respiratory	Levy[28] Zanobetti[29] Pooled	280 (150–420)	$9.1 ($4.7–$13)
Acute Myocardial Infarction, Nonfatal	Mustafic et al.[30]	220 (130–310)	$20 ($11–$27)
	**Total**		**$29,000 ($2,300–$68,000)**

### Estimation of Costs

We use the IPM output to develop three partial equilibrium cost cases to compare with the partial equilibrium co-benefit estimates. The IPM runs were designed to simulate the electricity sector response to constraints on CO_2_ emissions by improving the operation of existing facilities, substituting to lower emitting technologies, and by investing in demand-side energy efficiency. The policy scenario we examine assumes that incentives are created for programmatic funding of energy efficiency. At the assumed cost and level of funding, energy efficiency contributes most of the mitigation that is achieved in the policy scenario we analyze[2].

Our measure of costs includes capital, operations and maintenance for generation and investments in energy efficiency and assumes a default real interest rate of 4.77% for all expenditures. The electricity system costs in the implementation year under the policy scenario reflect the difference from BAU in the annualized costs of investments made between the announcement of the policy and the implementation year, plus changes in operations and maintenance in the implementation year. The costs for capital and operations and maintenance are the same in each of the three cost cases because generation is the same. Uncertainty arises in how to account for the costs of energy efficiency, and we explore three options.

There are two main components to the costs of energy efficiency investments. The first, program spending, includes 18% for administration and 82% for investment and is incurred by the utility or some other entity. This cost is recovered through a charge on electricity bills. The second, participant cost (i.e. the matching contribution of the residential, industrial or commercial property owner where the energy efficiency investment occurs) we assume to be equal to the program investment of 82% of and additional to the total program costs. In our central cost case we assume the programmatic energy efficiency investment costs are annualized while participant costs are incurred in the present year (“overnight”). The lower bound cost case assumes that both program and participant costs after 2013 are annualized. The upper bound cost case is an extreme case that assumes that both program and participant costs are incurred overnight.

### Net Co-Benefit Calculation

We calculate annual net co-benefits in the implementation year as the difference between the value of co-benefits for the central estimate and 95% confidence intervals and costs for the three cases. Investments in energy efficiency in the policy scenarios begin to ramp up in 2013 providing accrued measures in place that contribute to reduced demand in the implementation year 2020. Hence the associated air quality benefits are not strictly due to investments in 2020. On the other hand, investments that year yield air quality co-benefits in the future. We report net co-benefits as a snapshot, comparing the co-benefits with investment costs in 2020, not counting the benefits that will continue to flow.

To reveal the spatial distribution of net co-benefits, we compare estimated costs with the value of health co-benefits by power supply region. For this analysis we use approximate state boundaries for the 14 IPM power supply regions. Additionally, to calculate the time it would take for health co-benefits to equal the program costs for an investment in the implementation year, we sum the annual co-benefits from our central cost case over time and compare this with the costs in the implementation year plus the remaining annual payments for subsequent years (without discounting) for that portion of costs that is not recovered overnight.

## Results and Discussion

### Magnitude of Co-benefits and Costs

The national total of the monetized health co-benefits in the implementation year 2020 is $29 billion 2010 USD (95% CI: $2.3 to $68 billion)(Table 1). Most of this value (99.8%) is associated with avoided mortality due to decreases in PM_2.5_ and ozone (Table 1); the remainder is derived from morbidity effects. Results below are in 2010 USD, unless otherwise noted.

Under the central cost case, the total cost in the implementation year is $17 billion. The estimated cost under the lower cost case in which all costs are annualized is -$450 million. Negative costs in the implementation year occur in this case because the program-driven expenditures on energy efficiency are spread out over time but yield immediate savings in generation-related costs. The savings continue in future years, so the negative costs apply for each year in the program. The estimate for the upper case in which all costs occur overnight is $39 billion. The higher costs in this case are due to the upfront loading of all energy efficiency costs.

The net co-benefits for the central estimate for health co-benefits and central cost case is $12 billion (95% CI: -$15 billion to $51 billion). Positive net co-benefits indicate that the value of the health co-benefits are greater than the costs of the policy scenario, without taking into account additional health benefits, ecosystem benefits (e.g., visibility, crop and tree productivity)[31], or climate change benefits. The net co-benefit under the lower cost case is $30 billion (95% CI: $2.7 billion to $69 billion). The net co-benefit under the upper cost case is -$10 billion (95% CI: -$37 billion to $29 billion); in this case the health co-benefits are less than the costs of the policy in that year.

### Spatial Distribution of Co-benefits and Costs

**Fig 1:**
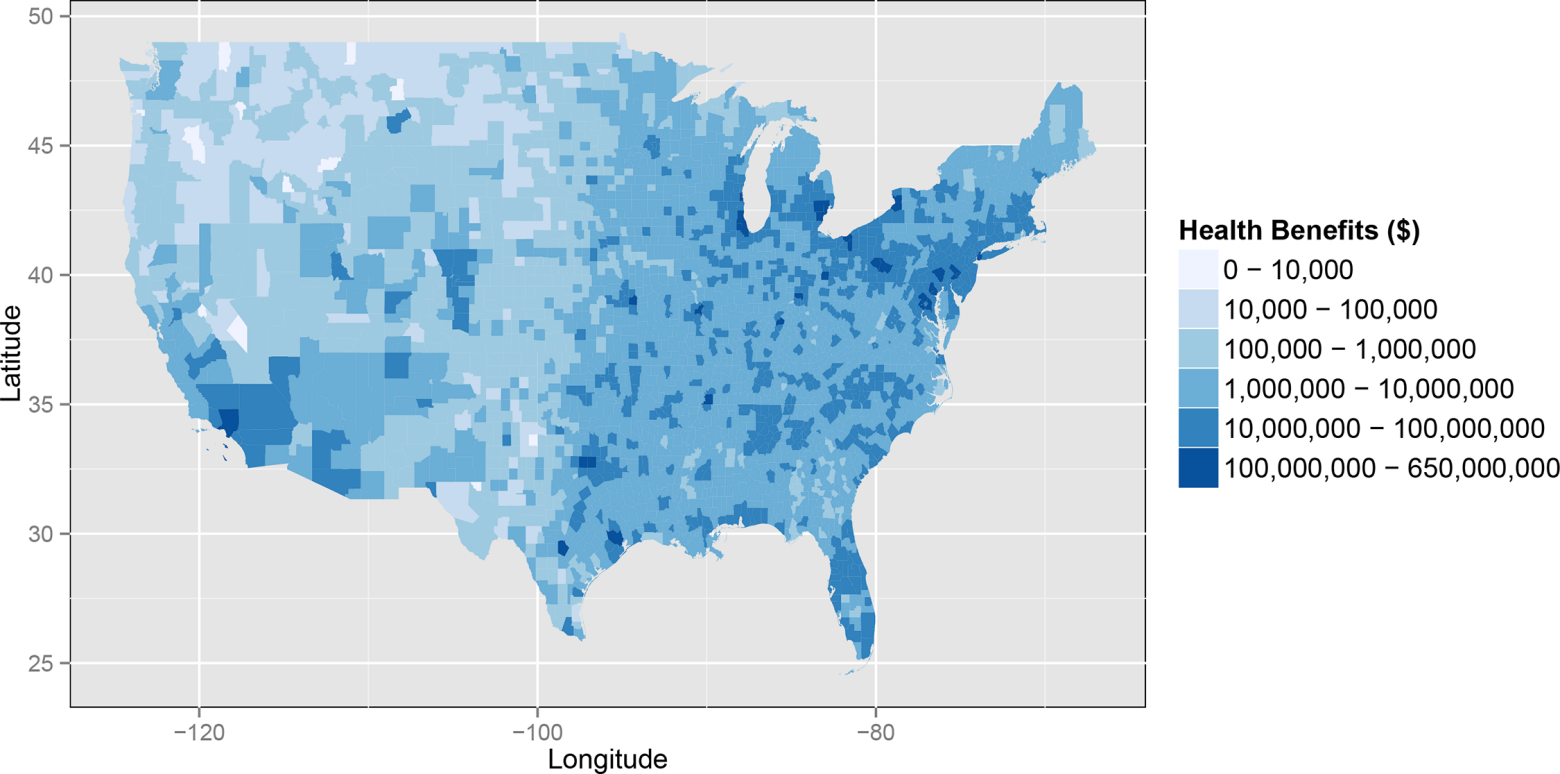
Total annual co-benefits of moderately stringent, highly flexible carbon standards in 2020 (2010 USD).

**Fig 2:**
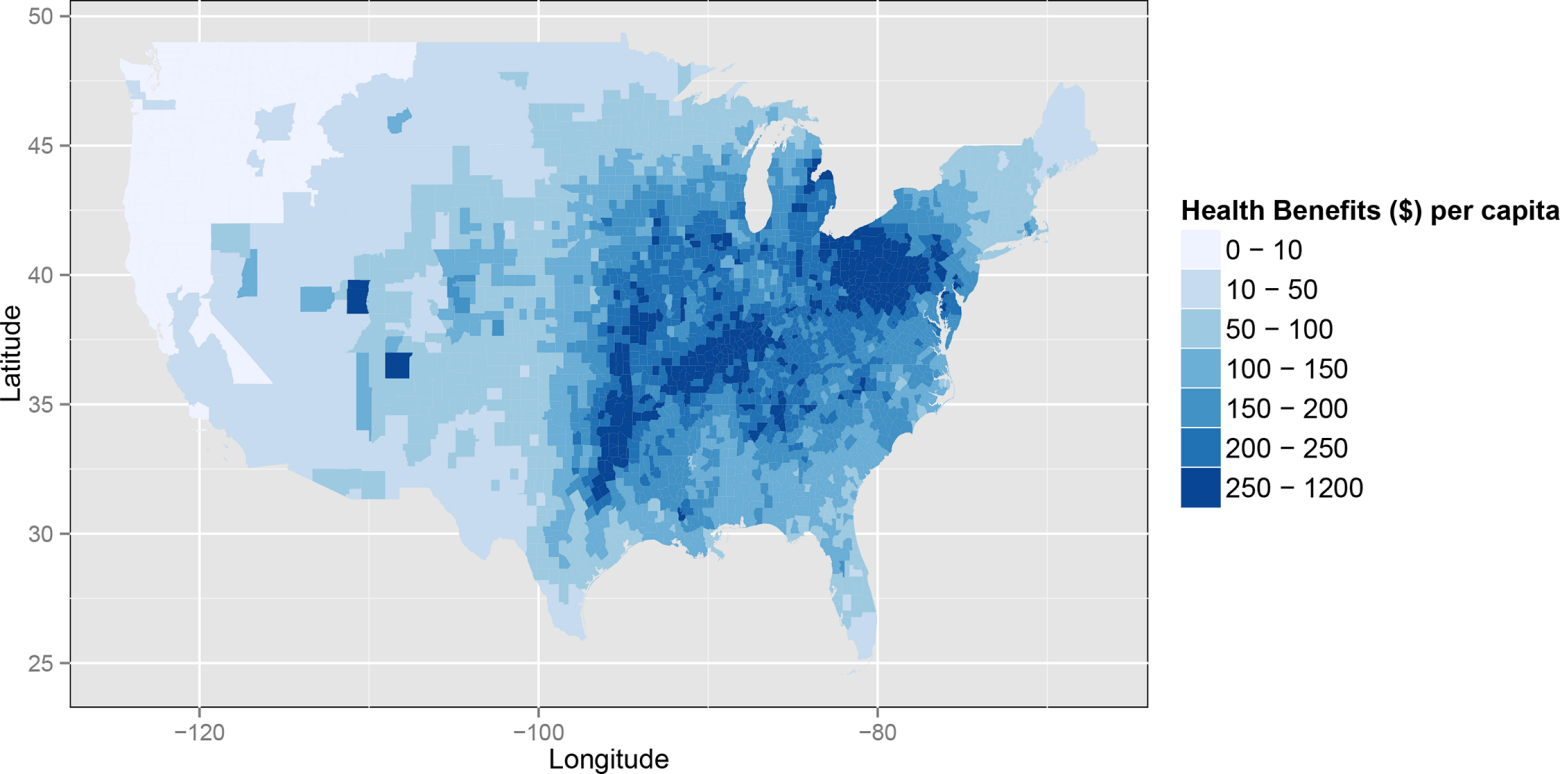
Annual co-benefits per capita for 18 to 99 year-olds under moderately stringent, highly flexible carbon standards in 2020 (2010 USD).

All counties of the continental U.S. receive annual co-benefits under the policy scenario in 2020 (Figs 1 and 2). Most counties gain at least $1 million in annual co-benefits, using our central estimate, and co-benefits are highest in the Northeast and Southwest U.S. (Figs 1 and 2). Health co-benefits per capita are greatest in Mid-Atlantic, Ohio River Valley, and South-Central regions of the U.S. (areas within the IPM regions PJME, PJMC, MISO, SERCC, SERCD, and ERCOT), with nearly every individual in these regions gaining at least $100 of co-benefits per year under the central estimate (Fig 2).

Central estimates of the annual co-benefits in the implementation year for each of the 14 IPM regions range from $5.6 billion in the Midwest (MISO and SERCG) to $57 million in the Pacific Northwest (PNW) (Table 2). The greatest health co-benefits occur in areas that have historically had a large amount of electricity generation from coal and are characterized by relatively poor air quality prior to 2020, and therefore receive large improvements in air quality under this scenario.

**Table 2:** Monetized value of annual co-benefits, costs, and net co-benefits by cost case for U.S. and IPM regions in 2020 (million 2010 USD). All values are calculated and then rounded to two significant figures, so net co-benefits may not sum perfectly.

			Lower cost case: All Costs Annualized		Central cost case: Annualized Program Costs, Overnight Consumer Costs		Upper cost case: All Costs Overnight	
IPM Region	States	Health Co-benefits (95% CI)	Cost	Net Co-Benefits (95% CI)	Cost	Net Co-Benefits (95% CI)	Cost	Net Co-Benefits (95% CI)
US	All lower 48 states	29,000 (2,300–68,000)	-450	30,000 (2,700–69,000)	17,000	12,000 (-15,000–51,000)	39,000	-10,000 (-37,000–29,000)
CALIFORNIA	CA	480 (37–1,100)	360	110 (-330–760)	1,400	-960 (-1,400–-310)	2,700	-2,300 (-2,700–-1,600)
ERCOT	TX	1,900 (150–4,500)	170	1,800 (-14–4,400)	1,800	100 (-1,700–2,700)	3,800	-1,900 (-3,700–690)
FRCC	FL	900 (71–2,100)	-140	1,000 (210–2,300)	960	-56 (-880–1,200)	2,300	-1,400 (-2,200–-170)
ISONE	ME, VT, NH, MA, CT, RI	880 (69–2,100)	220	660 (-150–1,900)	690	190 (-630–1,400)	1,300	-390 (-1,200–810)
MISO and SERCG	IN, MI, IL, WI, IA, MN, SD, ND	5,600 (440–13,000)	140	5,500 (290–13,000)	3,600	2,100 (-3,100–9,700)	7,800	-2,100 (-7,300–5,500)
NYISO	NY	1,600 (120–3,700)	110	1,400 (5.7–3,600)	610	950 (-490–3,100)	1,200	350 (-1,100–2,500)
OTHERWEST	WY, NV, UT, CO, AZ, NM	970 (80–2,300)	740	220 (-660–1,500)	1,800	-820 (-1,700–480)	3,100	-2,100 (-3,000–-800)
PJMC	OH, PA, WV	5,400 (420–13,000)	-1,600	7,100 (2,100–14,000)	310	5,100 (110–13,000)	2,700	2,700 (-2,300–10,000)
PJME	NJ, DE, MD, VA	3,000 (230–7,000)	890	2,100 (-660–6,100)	2,500	440 (-2,300–4,500)	4,500	-1,500 (-4,300–2,500)
PNW	WA, ID, MT, OR	57 (4.8–130)	320	-260 (-320–-190)	980	-920 (-970–-850)	1,800	-1,700 (-1,800–-1,600)
SERCC	NC, SC, GA, AL	1,700 (130–4,000)	-930	2,600 (1,100–4,900)	-26	1,700 (160–4,000)	1,100	610 (-950–2,900)
SERCD	AR, LA, MS	1,300 (100–3,000)	-120	1,400 (220–3,100)	790	490 (-690–2,200)	1,900	-620 (-1,800–1,100)
SERCSE		3,300 (260–7,700)	-570	3,900 (830–8,300)	1,500	1,800 (-1,200–6,200)	4,000	-760 (-3,800–3,700)
SPP	NE, KS, MO, OK	2,000 (160–4,700)	11	2,000 (150–4,700)	450	1,600 (-290–4,300)	990	1,000 (-830–3,700)

Costs in 2020 for the IPM regions range from $7.8 billion for the Midwest (MISO and SERCG) under the upper cost case to $-1.6 billion for the central mid-Atlantic region (PJMC) under the lower cost case (Table 2). Regions with high baseline emissions and large projected emissions reductions tend to have the highest costs–MISO, SERCG, PJME, and OTHERWEST. Generally, the Mid-Atlantic (PJMC), the Southeast (SERCC and SERCSE), the Southern Power Pool (SPP) and New York (NYISO) had lower costs.

**Fig 3:**
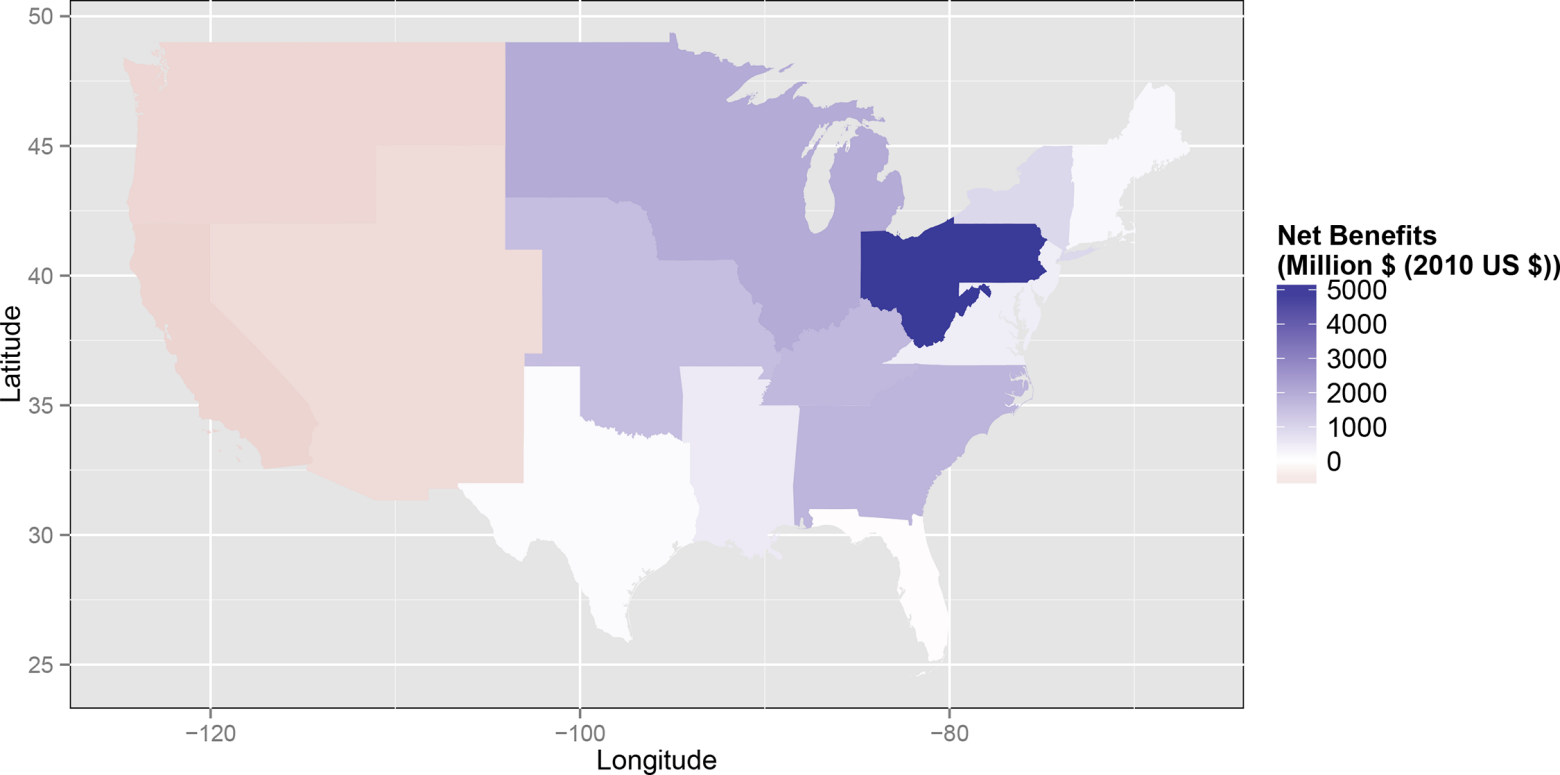
Net benefits by IPM Region for a moderately stringent, highly flexible carbon standard in 2020 (2010 USD) using central estimates for both cost and health co-benefits.

Using the central estimates for health co-benefits, the regional net co-benefits (i.e. value of co-benefits minus costs) in 2020 range from a high of $7.1 billion in the central mid-Atlantic (PJMC) region under the lower cost case to a low of $-2.3 billion in California under the upper cost case (Table 2, Fig 3). The results show that under the central cost case the 2020 net co-benefits are positive in 10 out of 14 regions (Table 2, Fig 3). For the lower cost case, the 2020 net co-benefits are positive in 13 regions of 14 regions (Table 2, Fig 3). Notably, even in the upper cost case, there are positive net co-benefits in 2020 in four out of 14 regions (Table 2, Fig 3 and S1 Fig). Further, co-benefits continue to accumulate over time, and so do costs in the central and low cost case. On an undiscounted basis for co-benefits, using our central cost case, the value of annual health co-benefits outweigh costs in FRCC in less than 2 years, they outweigh costs in OTHERWEST in less than 3 years, and they outweigh costs in California within 5 years of the implementation year. However, the co-benefits do not outweigh costs in the Pacific Northwest within the program period. Notably, this payback period is based on the limited health co-benefits included here and does not incorporate future avoided costs from CO_2_ reductions.

### Uncertainty in Co-benefits and Costs

The health co-benefits from the policy scenario analyzed here represent just a subset of the total health co-benefits that would be expected due to reductions in PM_2.5_, ozone, and other air pollutants. Specifically, we did not include co-benefits of avoided asthma[16], stroke[17], autism[18], and other health endpoints[16,32]. We also relied on large cohort studies that do not include impacts to people younger than 18 years. Finally, we did not include the benefits associated with lower emissions of air toxics, such as mercury, cadmium, carbon monoxide, and polycyclic aromatic hydrocarbons[19], and assumed that all particle types had the same toxicity[28].

The co-benefit estimates do not include the direct health benefits due to the mitigation of climate change, such as fewer heat-related illnesses[33] or a deterioration of air quality associated with climate change[20]. In addition, known benefits to natural resources, such as visibility improvements[34] and increased crop[35] and timber productivity [31,36]associated with lower ozone are not included.

The valuation of climate benefits is less advanced than the valuation of health co-benefits, but the literature is developing rapidly. The U.S. government has identified a central case value of $40 (2010 USD) of benefits in 2020 per short ton of CO_2_ emissions reduction, accounting for benefits that accrue domestically and internationally[37]. The moderately stringent, highly flexible policy scenario we evaluate results in reductions of 531.2 million short tons[2], which is approximately equivalent to $21.2 billion in direct climate benefits, using the U.S. regulatory social cost of carbon[38]. Therefore, the total estimated benefits for the scenario total approximately $50 billion per year in 2020 when both the estimated health co-benefits and social cost of carbon are included. This may be a conservative estimate for the value of climate damages since this value is lower than many recently published values for the social cost of carbon[39–41]. However, the implications of other values for the social cost of carbon can be explored by linearly scaling[39–41]. This result is consistent with previous co-benefit studies on policies affecting electricity generation[7,8,10,11,42–44].

The three cost cases presented here demonstrate that economic assumptions strongly influence net benefit results. Most of the range in net benefits, holding health co-benefits constant at the central estimate, is attributable to how the cost of energy efficiency is handled. Therefore, it is important to consider the plausibility of each cost case. A substantial literature critically questions whether and why potentially cost-effective opportunities for energy efficiency investments may go unrealized[45–48]. Nonetheless, empirical evidence from many programs suggests program spending on energy efficiency may have negative costs, even before considering environmental benefits[49,50]. In some cases investments in energy efficiency can actually reduce total system costs, even after accounting for the participant cost.

The power plant carbon standards policy scenario evaluated here will deliver a relatively consistent stream of health co-benefits over time, compared to no carbon standard, but the estimated stream of costs varies over time depending on economic assumptions. The model assumes spending on energy efficiency begins in 2013 and increases through 2025. The co-benefits of this spending accrue for many years after the investment is made, so the net co-benefits are not yet at their maximum level in 2020. Therefore, the comparison of co-benefits with costs in 2020 represents lower net benefits than what we would expect when the program is fully implemented in 2030.

This analysis is based on a reference case from the year 2013 based on the 2013 Annual Energy Outlook[13] and a 2014 policy case. Since that time, energy demand, renewable energy penetration, renewable energy and efficiency costs, and projections have changed, and the Clean Power Plan has also been finalized. While this may limit the ability of the scenario here to represent the final version of the Clean Power Plan, we expect the relationships between benefits and costs, and the geographical trends to remain similar. Finally, the results we present here are only partial equilibrium estimates of costs and air quality co-benefits. Additional costs and benefits that would be identified in a general equilibrium framework could be substantial but may be offsetting in the balancing of costs and co-benefits [51].

### Policy Implications

We found that for a moderately stringent, highly flexible policy scenario similar to the final U.S. Clean Power Plan, the monetized value of health co-benefits alone exceed estimated costs for the U.S. by $17 billion per year in 2020. When the social cost of carbon is included, the benefits increase from $29 billion to $50 billion with national net benefits of $38 billion per year in 2020. The central cost case assumes annualized program costs and overnight consumer participant costs for energy efficiency.

We also found that the estimated costs of a policy scenario for power plant carbon standards that is similar to the Clean Power Plan vary substantially across regions and under different economic assumptions. At a regional scale, the monetized value of the health co-benefits exceed costs in ten of 14 power regions in 2020 in the central estimate of health co-benefits and the central cost case. Further, annual co-benefits in excess of costs continue to accumulate after the implementation year. Consequently even in the high cost case, where only four power regions have positive co-benefits in the implementation year undiscounted co-benefits will exceed costs within six years for all regions except the Pacific Northwest. Therefore, even after accounting for uncertainty for cost recovery we anticipate that the value of health co-benefits will exceed costs under the central cost case in all but one of the power regions in the U.S. by the time the standards are fully implemented in 2030.

As this and other studies demonstrate, the health co-benefits gained from air quality improvements associated with climate mitigation policies can be large, widespread, and occur nearly immediately once emissions reductions are realized [2,44,52]. As such, health co-benefits can offset costs and provide an important additional motivation for policies that target greenhouse gas emissions, including the U.S. Federal Clean Power Plan.

## Supporting Information

S1 Fig Monetized value of net co-benefits under three different cost cases and the central estimate of health co-benefits for 14 power regions (2010 USD) in the year 2020. S1a Fig represents the lower cost case; S1b represents the central cost case; S1c represents the upper cost case.
(EPS)

S1 Table Health Impact Functions. (DOCX)

S2 Table Cost per case in U.S. (2010 USD). (DOCX)
