# Correction: An Analysis of Costs and Health Co-Benefits for a U.S. Power Plant Carbon Standard

**DOI:** 10.1371/journal.pone.0158792

**Published:** 2016-06-30

**Authors:** Jonathan J. Buonocore, Kathleen F. Lambert, Dallas Burtraw, Samantha Sekar, Charles T. Driscoll

There are numerical errors in the first two sentences of the “Policy Implications” heading under the Results and Discussion section. The correct sentences are: We found that for a moderately stringent, highly flexible policy scenario similar to the final U.S. Clean Power Plan, the monetized value of health co-benefits alone exceed estimated costs for the U.S. by $12 billion per year in 2020. When the social cost of carbon is included, the benefits increase from $29 billion to $50 billion with national net benefits of $33 billion per year in 2020.
