# Supplementary material for: An Analysis of Costs and Health Co-Benefits for a U.S. Power Plant Carbon Standard
Source: PLoS One. 2016 Jun 7;11(6):e0156308. doi: 10.1371/journal.pone.0156308 (PMC4896433; doi:10.1371/journal.pone.0156308)
Supplement: S1 Table — (DOCX) [file pone.0156308.s002.docx]

Table S1: Health Impact Functions

| Health Impact From: | Health Outcome | Pollutant | Beta (% risk increase / µg/m^3^ for PM_2.5_) (% risk increase / ppm for ozone) | Standard Error (% risk increase) |
| --- | --- | --- | --- | --- |
| Roman et al.^36^ | All-cause Mortality | PM_2.5_ | 1 | 0.4 |
| Levy^39^ Zanobetti^40^ Pooled | Respiratory Hospitalizations | PM_2.5_ | 0.11 | 0.027 |
| Levy^39^ Zanobetti^40^ Pooled | Cardiovascular Hospitalizations | PM_2.5_ | 0.094 | 0.015 |
| Mustafic et al.^41^ | Acute Non-fatal Myocardial Infarction (heart attack) | PM_2.5_ | 0.25 | 0.0536 |
| Jerrett et al.^37^ | Mortality from respiratory causes | Ozone | 0.39 | 0.13 |
| Ji et al.^38^ | Respiratory Hospitalizations | Ozone | 0.16 | 0.052 |
