# Supplementary material for: An Analysis of Costs and Health Co-Benefits for a U.S. Power Plant Carbon Standard
Source: PLoS One. 2016 Jun 7;11(6):e0156308. doi: 10.1371/journal.pone.0156308 (PMC4896433; doi:10.1371/journal.pone.0156308)
Supplement: S2 Table — (DOCX) [file pone.0156308.s003.docx]

Table S2: Cost per case in U.S. (2010$ USD)

| Source | Health Endpoint | Age Group | Medical Costs | Opportunity Costs | Total Central Estimate |
| --- | --- | --- | --- | --- | --- |
| Dockins *et al.* 2004 | Mortality | 25-99 | N/A | N/A | $6,985,000 |
| Eisenstein *et al.* 2001^17^ + Cropper and Krupnick (2000)^18^ | Acute Myocardial Infarction | 18-24 | $63,057 | $0 | $63,057 |
| Eisenstein *et al.* 2001^17^ + Cropper and Krupnick (2000)^18^ | Acute Myocardial Infarction | 25-44 | $63,057 | $10,871 | $73,928 |
| Eisenstein *et al.* 2001^17^ + Cropper and Krupnick (2000)^18^ | Acute Myocardial Infarction | 45-54 | $63,057 | $16,022 | $79,079 |
| Eisenstein *et al.* 2001^17^ + Cropper and Krupnick (2000)^18^ | Acute Myocardial Infarction | 55-64 | $63,057 | $92,612 | $155,668 |
| Eisenstein *et al.* 2001^17^ + Cropper and Krupnick (2000)^18^ | Acute Myocardial Infarction | 65-99 | $63,057 | $0 | $49.651 |
| HCUP 2007^19^ | Hospital Admissions, All Respiratory | 65-99 | $27,116 | N/A | $27,116 |
| HCUP 2007^19^ | Hospital Admission, All Cardiovas-cular | 65-99 | $32,314 | N/A | $32,314 |
